# Invited commentary: target trial emulation—a call for more widespread use

**DOI:** 10.1093/aje/kwae222

**Published:** 2024-07-25

**Authors:** Amanda Hyre Anderson

**Affiliations:** Department of Epidemiology, School of Public Health, University of Alabama at Birmingham, Birmingham, AL 35294, United States

**Keywords:** target trial emulation, observational studies, causal inference, bias

## Abstract

Causal inference methods intended for use with observational data have been widely available for decades, but barriers to their widespread adoption exist. These likely include lack of familiarity with several methodological techniques often used in combination in these investigations, such as inverse probability of treatment weighting and g-estimation, and the intensity of computational effort needed to employ these techniques. Even with these methods, critical design flaws undermine the ability to make valid causal inference in some studies. Identification of the need to explicitly pair study design elements with these causal inference methods led to development of a methodological approach recently termed *target trial emulation*. This approach requires that investigators define a hypothetical randomized trial, emulate that hypothetical protocol in assembling the cohort and defining study elements, and then conduct an analysis that attempts to mirror random treatment assignment. In the accompanying article by Heindel et al (*Am J Epidemiol*. 2025;194(3):651-658), the authors successfully emulate a target trial of systemic heparin during arteriovenous fistula creation on short-term endpoints by utilizing data from 2 existing randomized trials with key confounders available. Target trial emulation provides a framework with which to promote valid inference and generate high-quality contributions to the literature, and its use should be expanded.

This article is linked to “Evaluating the effectiveness of systemic heparin during arteriovenous fistula creation by emulating a target trial” (https://doi.org/10.1093/aje/kwae098).

***Editor’s note:** The opinions expressed in this article are those of the author and do not necessarily reflect the views of the* American Journal of Epidemiology.

## Introduction

Causal interpretation of observational analyses is fraught with challenges and requires careful consideration of design elements, data utilization, and statistical approaches to eliminate bias and appropriately emulate the gold standard—a randomized trial. Statistical methods to support causal inference have been widely available for decades,^1^^‑^^3^ but barriers to their widespread use exist. These likely include lack of familiarity with these methodological techniques, such as inverse probability of treatment weighting, censoring weights, weight stabilization, and g-estimation modeling, and the intensity of computational effort needed to employ these techniques. Many published investigations using these methods have focused on addressing the specific methodological challenge of time-dependent confounding.^3^^‑^^6^ Separating and independently handling the intermediate (ie, causal) and confounding (ie, bias) components of the effect of individual variables over time was a noteworthy and necessary advancement in causal inference methods. However, adoption of causal modeling alone is insufficient to address all sources of bias that prevent valid causal interpretation in observational studies.

## The keystone study design

Even with the use of causal modeling, critical design flaws often undermine the ability to make valid causal inference.^7^ Common potential sources of bias in observational studies need to be addressed in order to mimic a randomized trial. The majority of these threats to validity relate to elements of study design. Once a causal study question has been posed, design elements, including eligibility criteria, treatment and outcome definitions, confounders, censoring events, time zero, and a statistical plan, need to be carefully considered and clearly articulated.

In an observational setting, treatment is not allocated randomly. This design process must include a thorough delineation of factors that may be indicators for treatment or lack thereof. These factors, either discrete or time-varying depending on the treatment definition, must be included in the defined set of confounders to allow this “hypothetical randomized trial”^7^ to truly emulate a randomized trial. Compared with a randomized trial protocol, the protocol for the hypothetical randomized trial will always need to be more complex and include additional factors that address the lack of randomization and an artifactual time zero. Similar to widely used new-user study designs, timing of initiation of treatment, depletion of susceptible subjects, and immortal time bias additionally require consideration.^7^^‑^^9^ Finally, eligibility determinations need to be made using only data available at the hypothetical time zero, as would be done in the actual randomized study.

## Pairing design with statistical methods: target trial emulation

Identification of the need to explicitly pair study design elements with these causal inference methods led to development of a methodological approach recently termed *target trial emulation*.^7^^,^^8^^,^^10^^,^^11^ This approach requires that investigators define a hypothetical randomized trial, emulate that hypothetical protocol in assembling the cohort and defining study elements, and then finally conduct an analysis that attempts to mirror random treatment assignment. Examples of the use of causal modeling, including marginal structural models with existing observational data, are increasing, but most lack the identification of a target trial framework and thus do not fully emulate a trial. Emulating a target trial, using either existing data from randomized trials on nonrandomized treatments, observational studies, or large administrative claims or electronic medical record (EMR) datasets, involves more than just employing causal statistical methods and interpretation. It requires the combination of a target trial design, definition of a trial time zero, use of appropriate statistical methods, and a causal interpretation.

## A cautionary tale: without appropriate data and adjustment, emulated target trials will be invalid

Caution must be employed in selecting and analyzing existing data in an emulated target trial. With the detailed hypothetical randomized trial protocol in hand, investigators must determine whether a sufficient data source is available to attempt the target trial. Existing randomized trial datasets with information on nonrandomized treatments may have the benefit of a clear treatment and time zero but have relatively limited availability of key confounding factors, which prevents emulation of random treatment allocation. Prospective observational study data may be more exhaustive with regard to inclusion of confounders but may not capture necessary granularity or specificity regarding treatment and its timing. Neither of these study types may be sized large enough to adequately power the investigation. Finally, large claims or EMR datasets may overcome statistical power issues but introduce their own challenges with regard to incomplete data, lack of standardized data collection and variable definitions, and missing confounding factors. Regardless of study type, attention should be paid to the suitability of the dataset for the conduct of the target trial, and the benefits of using a specific existing data source should be weighed against the bias that remains (ie, the extent to which portions of the hypothetical randomized trial protocol must be abandoned or altered).

## A successful example of target trial emulation: the study by Heindel et al

Two international, multicenter randomized trials conducted between 2014 and 2019 sought to compare vonapanitase topically applied to the artery and vein for 10 minutes immediately after radiocephalic arteriovenous fistula creation versus placebo.^12^^,^^13^ For the PATENCY-1 trial, the primary endpoint was primary patency (fistula survival without thrombosis or corrective procedure), and for the PATENCY-2 trial the endpoints were fistula use for hemodialysis and secondary patency (fistula survival without abandonment). Pooled data on the nonrandomized treatment of systemic heparin and key confounders from these 2 randomized trials were used in the study by Heindel et al^14^ to emulate a target pragmatic trial of the effect of systemic heparin use during arteriovenous fistula creation on short-term endpoints of thrombosis and bleeding given the lack of evidence and clinical practice guidelines to inform surgeons performing arteriovenous access surgeries. This cohort study with prospectively collected data included 914 patients, 61% of whom received systemic heparin during arteriovenous fistula creation. The authors reported no difference in the risk of bleeding and lower absolute and relative risks of access thrombosis (risk ratio = 0.72; 95% CI, 0.50-0.98) when comparing treatment groups at 14 days postoperatively.^14^ Their final interpretation was that the study supported weak causal evidence for use of systemic heparin during arteriovenous fistula creation.^14^

The success of the study by Heindel et al relies on the constellation of several factors. First, the question posed lends itself to this framework. There is clinical equipoise regarding the systemic use of heparin during creation of an arteriovenous fistula, and the target trial retains simplicity given that the treatment is discrete. Second, a hypothetical trial is clearly defined. The authors describe salient design elements for their target trial, including eligibility criteria, treatment definition, time zero, primary effectiveness and safety outcomes, secondary outcomes, and statistical methods for emulating random treatment allocation and accounting for loss to follow-up.^14^ Specifically, randomization was emulated using inverse probability of treatment weighting and treatment propensity scores; selection bias was addressed using inverse probability of censoring weighting and censoring propensity scores; and marginal risks were calculated using inverse-probability–weighted marginal structural models. Third, the authors identified an appropriate set of existing data in which they could emulate this target trial and account for important confounders. The treatment was unambiguous, as was time zero. Due to the nature of the study question and the data on hand, this study was also spared from immortal time bias and depletion of susceptible subjects.

## The case for more emulated target trials

With enhanced funding-agency data-sharing mandates, expansion and underutilization of data repositories, and the increasing availability of large claims and EMR datasets, target trial emulation is becoming more and more practical. In addition, published successful examples of target trials provide a road map to other investigators who wish to embark anew on this path.^14^^‑^^17^ Importantly, clear direction has been provided to engage in these multistep investigations that (1) articulate a causal study question and design a hypothetical randomized trial protocol with which to address it, (2) emulate the target trial with an appropriate data source and set of statistical approaches, and (3) provide a balanced causal interpretation.^7^^,^^10^^,^^11^^,^^18^^,^^19^ For investigators hesitant to engage in g-estimation and marginal structural modeling, the positive message is that several statistical techniques, including the more approachable propensity score paired with standard modeling approaches, can be sufficient depending on one’s treatment definition and timing.^7^

Target trial emulation provides a framework for engaging in valid causal inference using observational data. It focuses us on the elimination of biases that plague traditional observational study types and stresses the importance of good study design paired with appropriate statistical approaches. While we should always tread with caution when making causal interpretations from observational data, this framework gives us sufficient structure and conceptual guidance to promote valid inference and make high-quality contributions to the published literature.
